# Exploiting *in situ* NMR to monitor the formation of a metal–organic framework†

**DOI:** 10.1039/d0sc04892e

**Published:** 2020-11-20

**Authors:** Corey L. Jones, Colan E. Hughes, Hamish H.-M. Yeung, Alison Paul, Kenneth. D. M. Harris, Timothy L. Easun

**Affiliations:** School of Chemistry, Cardiff University Main Building, Park Place Cardiff CF10 3AT UK HarrisKDM@cardiff.ac.uk EasunTL@cardiff.ac.uk; School of Chemistry, University of Birmingham Edgbaston Birmingham B15 2TT UK

## Abstract

The formation processes of metal–organic frameworks are becoming more widely researched using *in situ* techniques, although there remains a scarcity of NMR studies in this field. In this work, the synthesis of framework MFM-500(Ni) has been investigated using an *in situ* NMR strategy that provides information on the time-evolution of the reaction and crystallization process. In our *in situ* NMR study of MFM-500(Ni) formation, liquid-phase ^1^H NMR data recorded as a function of time at fixed temperatures (between 60 and 100 °C) afford qualitative information on the solution-phase processes and quantitative information on the kinetics of crystallization, allowing the activation energies for nucleation (61.4 ± 9.7 kJ mol^−1^) and growth (72.9 ± 8.6 kJ mol^−1^) to be determined. *Ex situ* small-angle X-ray scattering studies (at 80 °C) provide complementary nanoscale information on the rapid self-assembly prior to MOF crystallization and *in situ* powder X-ray diffraction confirms that the only crystalline phase present during the reaction (at 90 °C) is phase-pure MFM-500(Ni). This work demonstrates that *in situ* NMR experiments can shed new light on MOF synthesis, opening up the technique to provide better understanding of how MOFs are formed.

## Abstract 2

A new *in situ* NMR methodology for studying the formation processes of MOFs is reported, supported by SAXS and PXRD experiments. Synthesis of a phosphonate-based MOF is described, from molecular aggregation through to nucleation and crystallisation.

## Graphical abstract

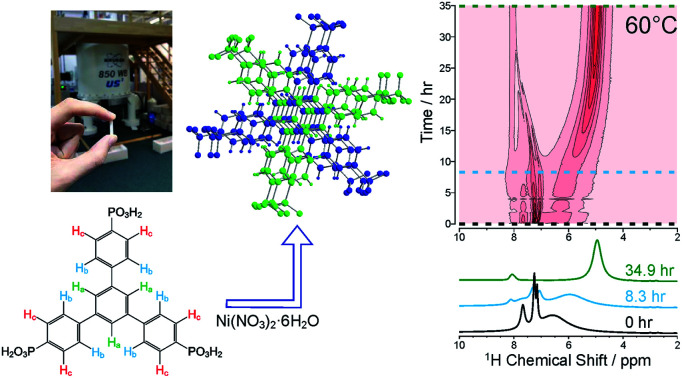

## Introduction

Metal–organic framework (MOF) materials are widely studied and have many applications in areas ranging from gas storage and separation^1–3^ to catalysis^4^ and chemical sensors.^5–7^ However, mechanistic aspects of MOF formation remain relatively understudied, with the majority of structural information obtained *post hoc*. Van Vleet *et al.* have reviewed the application of *in situ* techniques to monitor nucleation and growth of MOFs,^8^ including X-ray diffraction^9,10^ and other X-ray scattering techniques,^11–13^ while Cheetham *et al.* have described progress over the past 20 years in understanding the parameters that control crystallization of MOFs in solution.^14^ Although pre-nucleation and pre-equilibrium species have been shown to play a critical role in MOF formation reactions,^15^ the majority of studies have focused on nucleation and subsequent crystal growth.

In the last few years, Wu and co-workers have shown that it is possible to gain high-quality structural information from *in situ* synchrotron XRD measurements on a range of reactions, providing significant new insights into the time evolution of post-nucleation stages of MOF crystallization.^16–19^ Another example of this approach by Polyzoidis *et al.* detailed the formation of ZIF-8,^20^ while Zahn *et al.* used *in situ* energy dispersive X-ray diffraction to study the coordination-modulated formation of zirconium fumarate MOFs.^21^ Recently, X-ray scattering techniques have been combined with computational studies to determine the factors that control the nucleation and growth parameters in the polymerisation of 2D covalent organic frameworks (COFs).^22^

Microscopy techniques, including liquid cell transmission electron microscopy (LCTEM)^23^ and atomic force microscopy (AFM),^24,25^ have also become popular in investigating crystal growth mechanisms. These techniques can be extremely useful in combination with spectroscopic methods and X-ray scattering experiments, allowing multiple length scales of the MOF crystallization process to be probed.

To date, however, NMR spectroscopy has not been widely used to study the evolution of MOF syntheses. Nevertheless, solid-state NMR is a valuable technique for characterization of various aspects of MOF materials post-synthesis,^26,27^ including host–guest interactions, framework motion, and guest diffusion.^28,29^ Examples include the use of ^129^Xe NMR to identify interactions between frameworks and adsorbed guest molecules in an activated sample of UMCM-1,^30^ and studies of the diffusion of CO_2_ guest molecules within the pores of MOF-74-Mg.^31,32^ Notably, these methods all report the post-synthetic behaviour of MOFs.

In recent years, there has been progress in the development of techniques to monitor the time-evolution of crystallization of organic materials from solution using *in situ* NMR spectroscopy,^33^ both by the application of solid-state NMR measurements^34,35^ and by combined liquid-state and solid-state NMR measurements^36,37^ (the “CLASSIC” NMR technique). The CLASSIC NMR strategy, in particular, yields information simultaneously on the time-dependent changes that occur in the liquid phase (*e.g.*, changes in molecular aggregation and speciation) and in the solid phase (*e.g.*, changes in the polymorphic identity of the solid phase and the amount of solid produced) during crystallization from solution. In such experiments, the use of a high-field solid-state NMR spectrometer is essential to allow monitoring of both the liquid phase and the solid phase (we note that, if a traditional liquid-state NMR spectrometer were used to record liquid-state NMR data in a crystallizing system, the formation of the solid product would render shimming impossible to maintain). In addition to the application of these *in situ* NMR strategies to study organic crystallization systems, they are also a potentially powerful approach to gain new insights into MOF formation processes, including the nature of the initial liquid-phase reaction system and mechanistic aspects of the formation of the solid product.

Herein, we exploit this type of *in situ* NMR methodology (carried out using a high-field solid-state NMR spectrometer) to monitor the time-dependent changes that occur in a reaction system during MOF formation. The proton-conducting nickel–phosphonate MOF material MFM-500(Ni), first synthesized by Pili *et al.*,^38^ was chosen for this study as it provides the opportunity to measure both ^1^H and ^31^P NMR spectra and as the metal sites in the MOF material are non-paramagnetic. By studying the MOF synthesis at several fixed temperatures, we demonstrate that quantitative kinetic information on the crystallization of MFM-500(Ni) can be obtained, particularly from the *in situ* liquid-phase ^1^H NMR data. Small-angle X-ray scattering and *in situ* X-ray diffraction measurements provide complementary insight to the mechanism deduced by NMR, and reveal that formation of the crystalline MOF is likely preceded by aggregation of the linker into cylindrical stacks in solution.

## Experimental

**Scheme 1:**
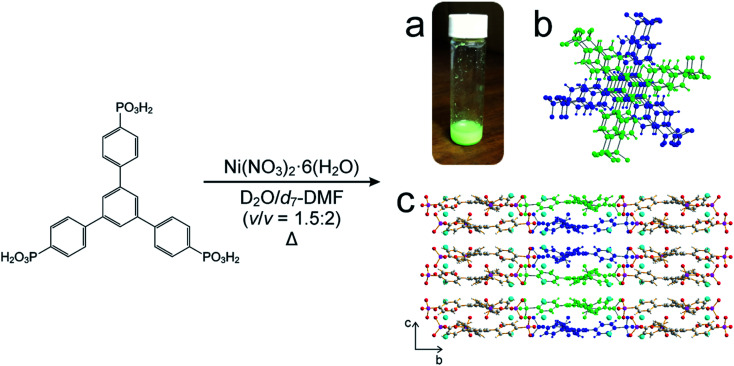
Adapted synthesis of MFM-500(Ni), in which 1,3,5-benzene-tri-*p*-phenyl phosphonic acid (BTPPA) and Ni(NO_3_)_2_·6H_2_O were reacted in deuterated solvent (D_2_O/d_7_-DMF) at the following temperatures: 60, 70, 80, 90, 100 °C. (a) Green crystalline solid product, (b) BTPPA linker dimer pairs in the crystal structure (see Fig. S7† for more detail), (c) structure of MFM-500(Ni) viewed along the *a*-axis. Central linkers are coloured blue and green as shown in (b) to show dimer paired stacks, with adjacent linkers shown using atom specific colours (H = white, C = grey, P = purple, O = red, Ni = cyan).

For our *in situ* NMR study of the synthesis of MFM-500(Ni), we simplified the previously reported synthesis^38,39^ by using only water and DMF as the solvent mixture (both of which were deuterated for the NMR measurements) and using increased concentrations of the reactants nickel nitrate [1.14 M] and 1,3,5-benzene-tri-*p*-phenylphosphonic acid (BTPPA) [0.57 M] (Scheme 1). Laboratory syntheses (see ESI† for details) and *in situ* NMR syntheses were carried out using different total volumes but identical concentrations and reactant ratios. In the *in situ* NMR experiments, the reaction solution (20 μL) was inserted into an NMR rotor and heated to the reaction temperature within the NMR spectrometer, with the experiment carried out at each of the following (fixed) temperatures: 60, 70, 80, 90, 100 °C. We note that the accessible temperature range of such experiments are limited by (i) the upper temperature limit of the NMR probe (<120 °C in our experiments) and (ii) pressure build-up in the sealed zirconia rotor. The *in situ* NMR strategy was implemented by recording a cycle of three different types of NMR measurement: (a) direct-excitation ^1^H NMR (to give the ^1^H NMR spectrum of the liquid phase), (b) direct-excitation ^31^P NMR without ^1^H decoupling (to give the ^31^P NMR spectrum of the liquid phase) and (c) direct-excitation ^31^P NMR with ^1^H decoupling (to give a ^31^P NMR spectrum containing contributions from both liquid and solid phases). This sequence of measurements was repeated throughout the duration of the experiment. The time to record one sequence of the three spectra was 7.1 min, representing the time-resolution of monitoring the MOF formation process in the *in situ* NMR experiment. The total duration of the experiment at each temperature was established from laboratory control experiments, and ranged from 4–36 h. For all NMR measurements, the MAS frequency was 12 kHz, the recycle delay was 3 s, and 90° pulses were used with nutation frequencies of 56 kHz (^1^H) and 42 kHz (^31^P). For ^1^H NMR and ^31^P NMR measurements without ^1^H decoupling, the acquisition comprised 4 scans. For ^31^P NMR measurements with ^1^H decoupling, the acquisition comprised 128 scans and ^1^H decoupling was carried out using SPINAL-64 (ref. 40) at a nutation frequency of 56 kHz. More details of the experimental procedures are reported in ESI.†

## Results and discussion

**Fig. 1:**
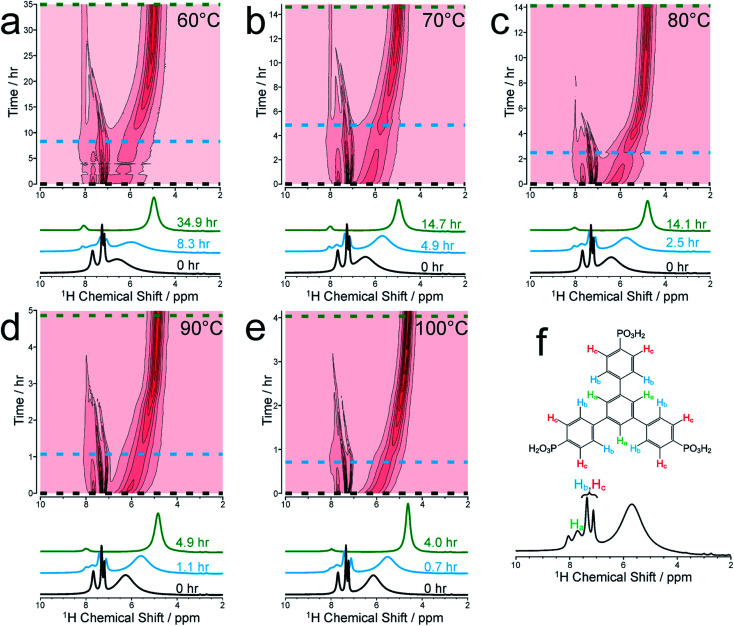
Intensity contour plots of the ^1^H NMR spectra recorded as a function of time in the *in situ* NMR study of MFM-500(Ni) synthesis, and individual spectra selected at specific times (indicated by horizontal dashed lines in the contour plots), at (a) 60 °C, (b) 70 °C, (c) 80 °C, (d) 90 °C and (e) 100 °C. Assignments of the three peaks due to aromatic ^1^H environments (denoted H_a_, H_b_ and H_c_) in the BTPPA linker are shown in (f). The spectra are shown without normalization.

We focus initially on the liquid-state ^1^H NMR spectra recorded in our *in situ* NMR studies, as they show well-defined evolution of several distinct resonances throughout the MOF formation process and provide more detailed information than the ^31^P NMR spectra (which are discussed below). Fig. 1a–e shows (as intensity contour plots) the time-evolution of the liquid-state ^1^H NMR spectrum at each temperature; three individual spectra from the beginning, middle and end of each experiment are also shown. At each temperature, the ^1^H NMR spectrum contains resonances in the range 7–8 ppm due to aromatic ^1^H environments (denoted H_a_, H_b_ and H_c_) in the linker, assigned in Fig. 1f (see section S2, Fig. S1† for NMR peak assignments). All three ^1^H signals in this range shift non-monotonically as a function of time during the reaction, initially to lower ppm and then to higher ppm (Fig. S2†).

To better understand the shifts of these ^1^H signals, ^1^H NMR spectra were measured for solutions containing just the BTPPA linker at room temperature in both d_6_-DMSO and in the reaction solvent mixture (D_2_O/d_7_-DMF) (Fig. S4 and S5† respectively), and also in the reaction solvent mixture at the temperatures used in the *in situ* NMR study (Fig. S6†). At room temperature in d_6_-DMSO, the ^1^H NMR peaks are sharp and well-resolved (Fig. S4†), but in the D_2_O/d_7_-DMF mixture (Fig. S5†) they are broader. Significantly, the peak due to ^1^H environments (H_a_) in the central aromatic ring of the linker is at lower ppm relative to the other aromatic peaks (H_b_ and H_c_), consistent with aggregation of the linker in the reaction solution. This interpretation is supported by the variable temperature ^1^H NMR spectra of the linker in the reaction solvent mixture (Fig. S6†); as temperature is increased, the peaks become increasingly well-resolved and shift to higher ppm, consistent with greater thermally-promoted disaggregation of the linker. We therefore propose that, at the start of each MOF synthesis, aggregation is much less prevalent than at room temperature. The positions of the aromatic proton peaks observed in our *in situ*^1^H NMR spectra are shown in Fig. S2.† The initial shift to lower ppm over time is consistent with initial aggregation of the linker (as per the control experiments just described and in agreement with conclusions from the SAXS experiments described below), but is also consistent with the direction of anticipated peak shifts on deprotonation of the BTPPA phosphonic acid groups,^41–44^ most notably observed in H_c_ at the *ortho* position with respect to the phosphonic acid substituents.^41,45–47^ Subsequent to these two combined effects, and on a more rapid timescale with increasing temperature, a marked swing in the other direction downfield to higher ppm is observed for all the aromatic protons, in line with metal coordination counteracting and exceeding the effects of deprotonation in particular. Significantly, these processes are not observable by simply monitoring nucleation and crystal growth by the other methods outlined in the Introduction above. The intensities of the ^1^H NMR peaks for the linker remain reasonably constant until nucleation and product precipitation begin, as discussed further below.

At each temperature, there is also a broad peak in the *in situ*^1^H NMR spectra, initially at ∼6.5 ppm but then shifting gradually towards ∼4.5 ppm and becoming sharper over the course of the reaction. The evolution of this peak is ascribed to a change in the solvent mixture during the reaction, resulting from liberation of (non-deuterated) water molecules from the nickel coordination sphere, which ultimately constitute a significant proportion of the final solvent, with simultaneous H/D exchange. At the end of the reaction, the nominal solvent ratio d_7_-DMF : D_2_O : H_2_O is approximately 4 : 3 : 1 (v/v/v). Solvent composition can cause changes in reaction mechanisms and crystallization pathways under hydrothermal conditions.^34,48^ However, in our syntheses of MFM-500(Ni), comparative experiments with deuterated and non-deuterated solvents carried out at matched temperatures both at 60 °C and at 80 °C have shown: (i) only MFM-500(Ni) is formed, and (ii) at each given temperature, visible formation of green microcrystalline product occurs on the same timescale irrespective of the level of deuteration of the solvent.

To corroborate the *in situ*^1^H NMR results, laboratory control experiments were carried out on a larger scale in which reaction solutions of identical concentration and reactant ratio were heated in screw-top vials at 60, 70, 80, 90 and 100 °C. For the experiment at 80 °C, ^1^H NMR and ^31^P{^1^H} NMR spectra were recorded *ex situ* for samples extracted periodically from the reaction solution, showing good agreement with the *in situ* NMR results (Fig. S8†). In the laboratory-control experiment at each temperature, the reaction occurred on a similar timescale to the corresponding *in situ* NMR experiment. Both sets of experiments produced a green crystalline material, which was shown by powder XRD to be phase-pure MFM-500(Ni) (Fig. S9†). In both the *in situ* NMR experiments and the laboratory-control experiments at all temperatures studied, this material was the only crystalline product observed.

**Fig. 2:**
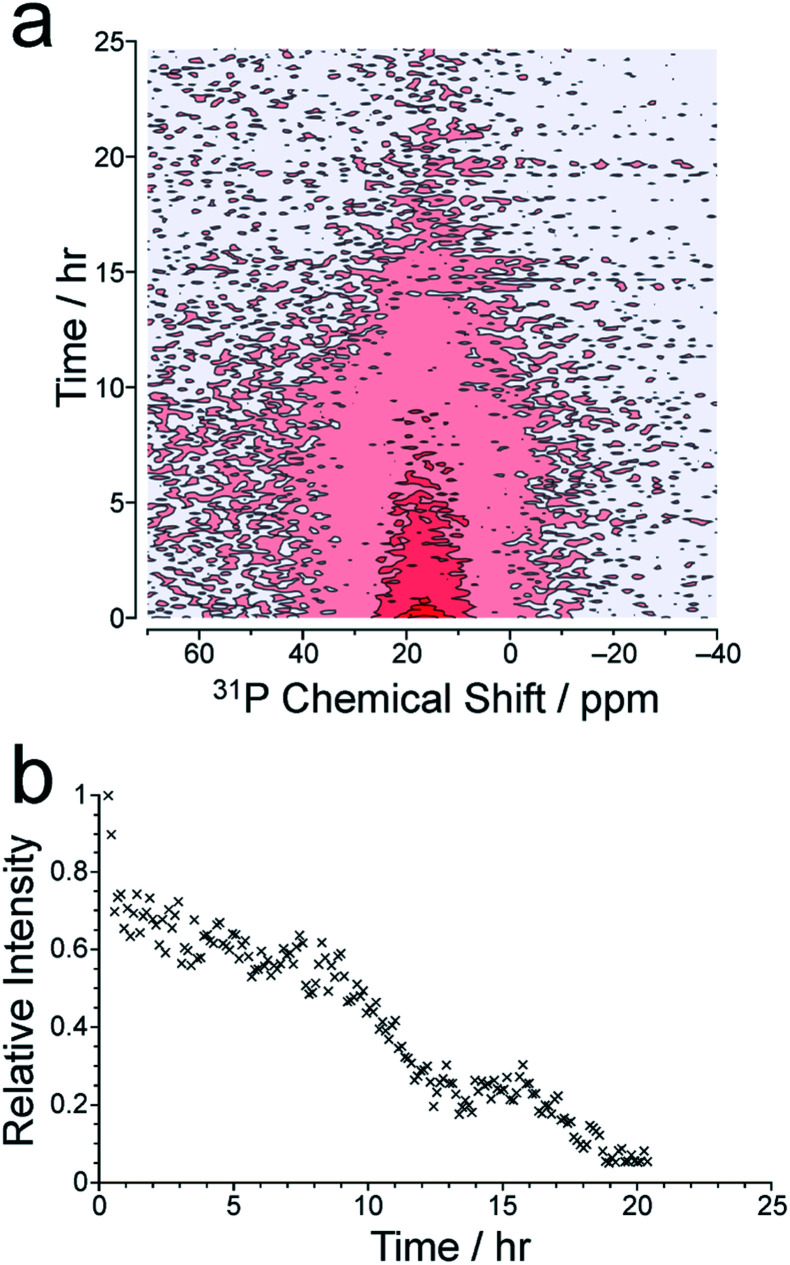
(a) Intensity contour plot showing the time-dependence of the *in situ*^31^P NMR spectrum (recorded without ^1^H decoupling) at 60 °C, representing the liquid-state ^31^P NMR signal. (b) Intensity *vs.* time plot for the liquid-state ^31^P NMR signal.

**Fig. 3:**
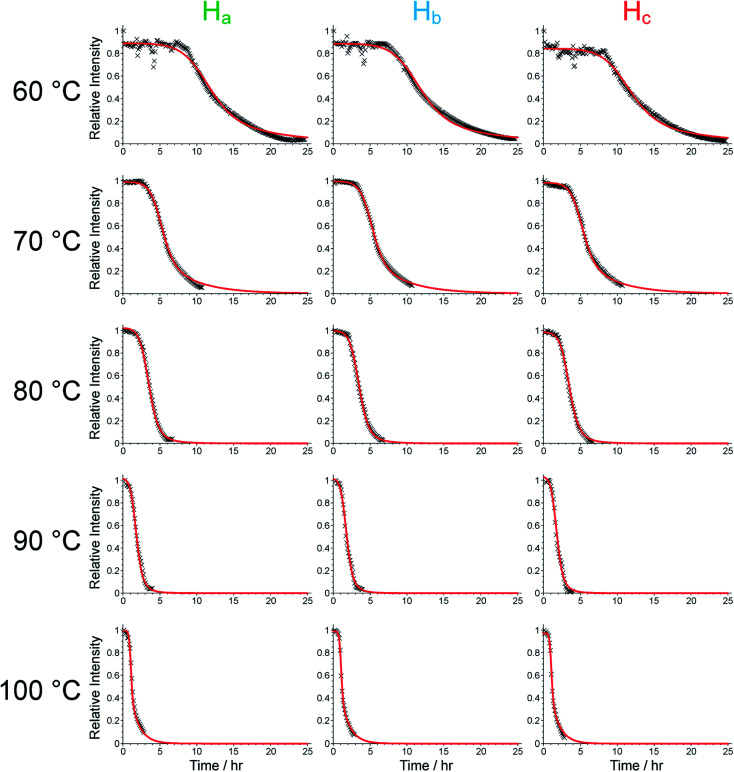
Intensity *vs.* time plots for the three aromatic peaks of the BTPPA linker obtained from the *in situ*^1^H NMR data, together with the fits obtained using the Gualtieri model (red lines). Oscillations in the peak intensities in the first ∼8 h of the experiment at 60 °C were caused by variations in the probe tuning.

We now consider the ^31^P NMR spectra recorded (with and without ^1^H decoupling) in the *in situ* NMR study. Fig. 2a shows an intensity contour plot of the *in situ*^31^P NMR spectra recorded without ^1^H decoupling (giving liquid-phase ^31^P NMR data) as a function of time during the MOF synthesis at 60 °C (and the first spectrum recorded in this experiment is shown in Fig. S10†). At this temperature, a single, broad peak is observed, and distinct ^31^P resonances from different linker environments are not resolved. The intensity of the signal decreases over the course of the experiment, consistent with loss of the linker from the solution phase. The intensity drops markedly from *ca*. 9 h (Fig. 2b) and continues to decay until *ca*. 20 h, comparable to the behaviour observed in the *in situ*^1^H NMR data at 60 °C (see Fig. 3).

The *in situ*^31^P NMR spectra recorded with ^1^H decoupling (which contain contributions from both liquid and solid phases) are uninformative regarding the formation of solid MFM-500(Ni) as the signal in these spectra remains broad and weak throughout the MOF formation process, possibly as a result of tuning/arcing problems experienced during measurement of these spectra. The first spectrum of this type recorded in the *in situ* NMR study at 60 °C is shown in Fig. S10.† At higher temperatures, the *in situ*^31^P NMR spectra recorded with ^1^H decoupling are also uninformative due to the broadness of the peaks and weakness of the signal. For comparison, the solid-state ^31^P NMR spectrum for a powder sample of MFM-500(Ni) prepared *ex situ*, recorded under analogous conditions to the *in situ*^31^P NMR spectra with ^1^H decoupling, is shown in Fig. S11.† While we have no clear explanation for the broadness of the solid-state ^31^P NMR spectra recorded with ^1^H decoupling in the *in situ* study, it is conceivable that some amount of paramagnetic Ni may be present in the solid phase formed during the reaction, possibly as a result of octahedral complex formation during synthesis or at defect sites in the framework.^49^

To analyse the formation of solid material during the syntheses, an *in situ* study of the reaction at 90 °C was carried out on beamline I12 at Diamond Light Source,^50^ recording the evolution of the powder XRD pattern as a function of time. An induction period of *ca*. 80 min is observed prior to formation of crystalline material of sufficient particle size to be observed by X-ray diffraction. The powder XRD data confirm that the first crystalline phase that appears is MFM-500(Ni), with no other crystalline phases observed at any stage of the reaction (Fig. S12†). Furthermore, the initial rise in the amount of MOF present is consistent with the rate of loss of the ^1^H NMR signal due to the BTPPA linker (Fig. 3). Unfortunately, the reaction kinetics could not be reliably determined from the powder XRD data as the high concentration of the reaction solution (used to mimic the conditions in our *in situ* NMR study) resulted in rapid formation of clumps of crystallites which tended to drop unpredictably out of the measurement region in the sample tube, leading to irregular drops in signal intensity (Fig. S13†). Instead, a quantitative kinetic analysis of the MOF formation process based on the results from our *in situ*^1^H NMR study is presented below; in this regard, we emphasize that a distinct advantage of the *in situ* NMR approach is that the data are measured for the whole sample volume throughout the experiment. The slightly longer induction time for product formation in the *in situ* powder XRD experiment can be attributed to the need to form crystalline particles of sufficient size to observe sharp peaks in the powder XRD data. In order to characterize the formation of smaller particulates that are potentially invisible to the powder XRD measurements, *ex situ* studies of MFM-500(Ni) formation were carried out using small-angle X-ray scattering (SAXS).

The timescale of the reaction at 90 °C, investigated by *in situ* powder XRD, was too fast for reliable SAXS measurements at this temperature. Instead, the reaction was carried out in the laboratory at 80 °C and SAXS data were recorded *ex situ* on samples extracted from the solution during the first 4 h of the reaction. Data analysis (Fig. S14 and S15†) shows the initial formation and growth of core–shell cylindrical particles, elongation of which accelerates at around 135 min. This observation is consistent with the period corresponding to the significant decrease in the intensity of the linker protons in the *in situ*^1^H NMR experiment from *ca.* 2.5 h onwards (Fig. 3). These data support the concept of pre-aggregation of the linkers in the reaction solution, as observed in the variable-temperature ^1^H NMR spectra of the linker described above (Fig. S4, S5 and S6†), with metal ions bridging these aggregates to form core–shell cylinders that grow throughout the initial period (see Section S16 of ESI† for more details). Furthermore, the crystal structure of MFM-500(Ni) contains ligand “dimers” in a staggered conformation with respect to the three arms of each linker around the central phenyl ring, which are then eclipsed to the next pair of dimers in the ligand “stack” along the *c*-axis, all of which are bridged by columns of metal ions (Fig. S7†).^38^ We propose that the cylindrical structures suggested by the SAXS experiment may be the precursors for these stacks (Fig. S16†).

We now focus on establishing quantitative information on the reaction kinetics from analysis of the time-dependence of the peak intensities in the *in situ* liquid-state ^1^H NMR spectra (Fig. 1a–e). At each of the five reaction temperatures, the decrease in peak intensities as a function of time for the three aromatic ^1^H resonances of the BTPPA linker (H_a_, H_b_ and H_c_) was successfully fitted using the two-stage model of Gualtieri.^51^ All peaks in each ^1^H NMR spectrum were fitted to Lorentzian lineshapes, with the five overlapping peaks at higher chemical shift fitted simultaneously (an example is shown Fig. S3†). Each Lorentzian was defined by chemical shift, linewidth and intensity, with polynomial functions used to fit the baseline of the spectrum. From such fitting of the ^1^H NMR spectra, the time-dependent intensities for the three linker peaks were established. These intensities were then normalized by scaling the values so that the highest intensity for each peak was set to unity (Fig. 3).

The Gualtieri model for nucleation and growth was used to fit our experimental data of peak intensities as a function of time (recalling that our measurements probe the decrease in peak intensities due to loss of the BTPPA reactant from the solution phase) using the following equation for the relative intensity of each peak as a function of time:
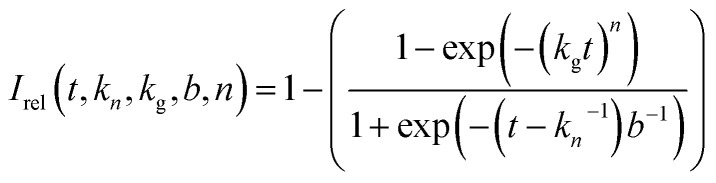
In this expression, *k*_*n*_ is the rate constant for nucleation, *b* is proportional to the standard deviation of the mean nucleation time (1/*k*_*n*_), *k*_g_ is the rate constant for crystal growth and *n* denotes the dimensionality of the growth process. At a given temperature, the time-dependence of all three peak intensities was fitted simultaneously using this model giving a single set of values of *k*_*n*_, *k*_g_ and *b*, with only a scaling factor (*s*_*j*_) varied independently for each peak (labelled *j* = 1, 2, 3). Thus, the intensity of peak *j* at temperature *T* and time *t* is given by:
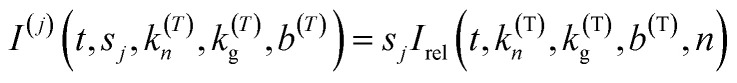


Consequently, the fitting of the data at each temperature involved only six fitted parameters: *s*_1_, *s*_2_, *s*_3_, *k*_*n*_, *k*_g_ and *b*. At each temperature, the fitting process was carried out for different (fixed) values of the parameter *n* (with *n* = 1, 2 or 3). In all cases, the best fits were obtained using *n* = 1. The values of *k*_*n*_, *k*_g_ and *b* obtained from the fitting process at each temperature are given in Table S1 (see section S5 of ESI).†

**Fig. 4:**
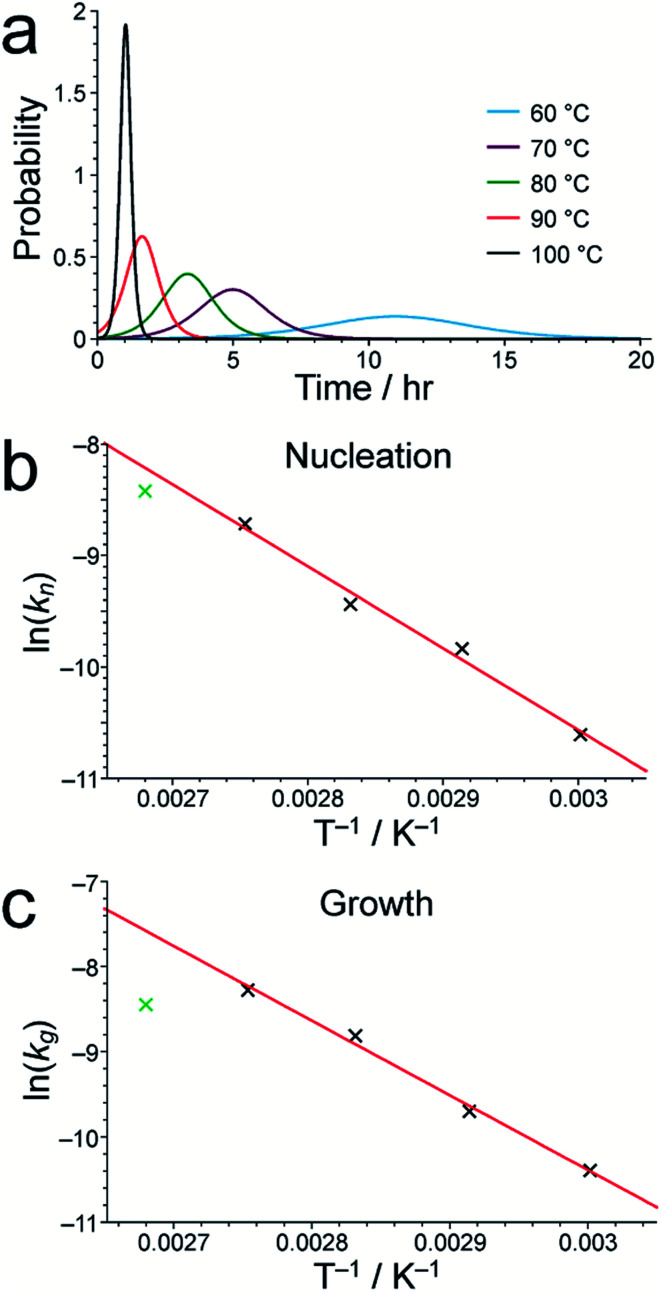
(a) The nucleation probability distribution for MFM-500(Ni) formation, obtained by fitting the *in situ*^1^H NMR data to a Gualtieri model at each temperature studied. Arrhenius plots for (b) nucleation and (c) growth of MFM-500(Ni) using the values of *k*_*n*_ and *k*_g_, respectively, determined from our *in situ*^1^H NMR data. In each case, the best-fit line was calculated using the data points for 60, 70, 80 and 90 °C, as discussed in the text.

As shown in Table S1,† the fitted parameters at 60, 70, 80 and 90 °C show the expected trends of increasing rate constants for nucleation (*k*_*n*_) and growth (*k*_g_) as temperature is increased, as well as a decrease in the standard deviation in the mean nucleation time (*b*) as temperature is increased. The probability distributions for nucleation as a function of time, determined from the kinetic parameters, are shown in Fig. 4a (see Section S5 of ESI†) and also exhibit the expected variation with temperature. The kinetic parameters determined at 60, 70, 80 and 90 °C are found to exhibit Arrhenius behaviour (Fig. 4b and c), allowing the activation energies for the nucleation and growth of MFM-500(Ni) to be determined as *E*_a_^(*n*)^ = 61.4 ± 9.7 kJ mol^−1^ and *E*_a_^(g)^ = 72.9 ± 8.6 kJ mol^−1^, respectively. These values are comparable to the activation energies of other single-phase forming reactions at similar temperatures determined using diffraction-based approaches.^52–56^ We note that the rate constant for growth (*k*_g_) determined at 100 °C is slightly lower than at 90 °C and clearly represents an outlier in the Arrhenius plot for the growth kinetics (Fig. 4c); for this reason, the results at 100 °C were omitted from the calculation of activation energies.^57^

## Conclusions

In summary, we have demonstrated the successful application of *in situ* NMR methodology to monitor the formation of MFM-500(Ni), yielding information on the time-evolution of the liquid phase prior to and during MOF formation. In particular, monitoring the time-dependent changes in ^1^H signal intensities allows activation parameters to be determined for the nucleation and crystal growth processes. This method extends the scope and capability of *in situ* monitoring of MOF syntheses, most significantly with regard to early-stage processes in the liquid phase, offering the possibility to gain new information that is typically unattainable by X-ray scattering techniques. This type of kinetic study of MOF formation, in which we rationalise the various processes involving the exchangeable and non-exchangeable ^1^H environments, is equally applicable to carboxylate-based MOFs. Since most reported MOF linkers contain ^1^H nuclei, the use of a solid-state NMR spectrometer with the *in situ*^1^H NMR methodology described in this work should be equally applicable to almost all linkers, whether they are carboxylate-based, imidazolate-based or other types of organic linker. Indeed, other spin-active nuclei could just as readily be probed to provide even greater depth of understanding of MOF formation processes, such as ^13^C NMR (*e.g.* studies of ^13^C-labelled carboxylate groups in linkers) or ^19^F NMR (*e.g.* for MOFs containing fluorinated linkers). Of course, the specifics of the NMR measurement technique will depend on the particular material of interest, but the same approach is widely applicable.

In this work, we found that *ex situ* SAXS and *in situ* powder XRD provide complementary and confirmatory information on the MFM-500(Ni) growth process, showing that formation of cylindrical linker-based aggregates precedes the appearance of crystalline MFM-500(Ni). Accessing precise chemical information about the early stages of MOF formation is still challenging, and future mechanistic investigations would do well to focus initially on the simplest systems available (*e.g.* single-solvent). Furthermore, at very early time points in the process when the concentrations of intermediates and product are very low, the application of new dynamic nuclear polarization (DNP) approaches may offer sensitivity enhancements that give new insights (as demonstrated recently in DNP solid-state NMR studies of crystallization of organic materials^58^). Such experiments could be based on existing, largely static studies to characterize MOFs post-synthesis.^26,59–61^ In the context of *in situ* studies of materials formation processes, there are disadvantages of DNP in comparison to the approach outlined in the present work as DNP requires the presence of a potentially non-innocent polarizing agent and as significant DNP intensity enhancement is observed only at low temperatures – making it less well suited to studying many MOF syntheses *in situ*.

The combined approach described in this work offers chemical insight into the dynamic solution-state supramolecular chemistry during MOF synthesis and highlights that the chemistry of the solution-state prior to formation of a long-range ordered product might be key to understanding MOF assembly. Further MOF syntheses are currently under investigation by the *in situ* NMR method, which can readily be extended to interrogate other NMR-active nuclei.

## Conflicts of interest

There are no conflicts to declare.

## Supplementary Material

SC-012-D0SC04892E-s001
