# Supplementary material for: Exploiting in situ NMR to monitor the formation of a metal–organic framework
Source: Chem Sci. 2020 Nov 20;12(4):1486–94. doi: 10.1039/d0sc04892e (PMC8179150; doi:10.1039/d0sc04892e)
Supplement: SC-012-D0SC04892E-s001 [file SC-012-D0SC04892E-s001.pdf]

Electronic Supplementary Material (ESI) for Chemical Science.
This journal is © The Royal Society of Chemistry 2020

Exploiting *in-situ* NMR to monitor the formation of a metal-organic framework.

Corey L. Jones, Colan E. Hughes, Hamish H.-M. Yeung, Alison Paul, Kenneth. D. M. Harris and Timothy L. Easun**

Supporting Information

Table of Contents

S1: Experimental Details 2
S2: ^{1}H NMR Peak Assignments in the *In-situ* NMR Study 5
S3: ^{1}H NMR Peak Shifts of BTPPA During the *In-situ* NMR study 6
S4: Peak Fitting of ^{1}H NMR Spectra Recorded during the *In-situ* NMR study 7
S5: Determination of Kinetic Information from the *In-situ* ^{1}H NMR Data 8
S6: NMR Spectra of BTPPA in d_6-DMSO 9
S7: NMR Spectra of BTPPA in D_2O/d_7-DMF 10
S8: Variable Temperature ^{1}H NMR Spectra of BTPPA in D_2O/d_7-DMF 11
S9: NMR Spectra Recorded *Ex-situ* During Laboratory Synthesis of MFM-500(Ni) 13
S10: Powder XRD Patterns of the Solid Products Obtained in MFM-500(Ni) Syntheses 14
S11: ^{31}P NMR Spectra Recorded at 60 °C in the *In-situ* NMR study 15
S12: Solid-state ^{31}P NMR Spectra for a Powder Sample of MFM-500(Ni) 17
S13: Powder XRD Patterns from *In-Situ* Powder XRD 18
S14: Attempts to Fit the *In-Situ* Powder XRD Data 19
S15: SAXS Data Recorded *Ex-situ* for Laboratory-synthesized MFM-500(Ni) 20
S16: Fitting of SAXS Data 21
References 24

S1: Experimental Details

Synthesis of BTPPA. 1,3,5-benzene-tri-*p*-phenylphosphonic acid was synthesized following a literature procedure.[1] A suspension of 1,3,5-tris(*p*-bromophenyl)benzene (2.0 g, 3.68 mmol), triisopropyl phosphite (12.0 mL, 52.3 mmol) and $Pd(PPh_3)_4$ (20 mg, 0.017 mmol) was heated at 120 °C for 3 hr. After 3 hr, additional triisopropyl phosphite (3.0 mL, 13.1 mmol) and $Pd(PPh_3)_4$ (20 mg, 0.017 mmol) were added. The mixture was heated to 205 °C for 2 - 3 days. Upon cooling, the crude product crystallized, and was then filtered and washed with hexane (60 mL) to give 1,3,5-[p-$C_6H_4P(O)(O_i\text{-}Pr)_2]_3C_6H_3$. This was then solubilized in HCl/H_2O (v/v = 2:1) and refluxed for 2 days. Upon cooling, a viscous oil formed. The supernatant was decanted off and the product was recrystallized from methanol. Yield: 92.8 %. **^{1}H NMR (500 MHz, d_6-DMSO):** δ = 8.01 (s, 3H), 7.99 (dd, J = 8.3, 3.2 Hz, 6H), 7.81 (dd, J = 12.6, 8.3 Hz, 6H). **^{31}P NMR (202 MHz, d_6-DMSO):** δ = 12.59 (s). **HRMS (ES$^-$):** m/z = 545.0343; calculated 545.0320 for $[C_{24}H_{20}O_9P_3]^-$. **IR:** ν_{max} / cm^{-1} = 1596, 1387, 1139, 999, 923, 818, 691, 535.

***In-situ* NMR Study of Synthesis of MFM-500(Ni).** Measurements were carried out on a Bruker AVANCE III NMR spectrometer at the UK High-Field Solid-State NMR Facility, based at the University of Warwick (^{1}H Larmor frequency, 850 MHz; ^{31}P Larmor frequency, 344 MHz; 4 mm HXY probe in double-resonance mode). BTPPA (0.025 g, 0.046 mmol) and $Ni(NO_3)_2\cdot 6H_2O$ (0.0265 g, 0.091 mmol) were dissolved in D_2O and d_7-DMF (80 µL, v/v = 1.5:2) at room temperature. An amount (20 µL) of the clear green solution was transferred to a Kel-F tube (sealed with a plug and screw), which was inserted into a zirconia rotor followed by a drive tip. The rotor was inserted into the spectrometer, spun at a MAS frequency of 12 kHz and temperature (calibrated against lead nitrate[2] and methanol[3–5]) was increased to the required value, taking into account the heating effect due to MAS (MAS at 12 kHz corresponds to a temperature increase of 13 °C). The temperatures studied were 60, 70, 80, 90 and 100 °C. Three types of NMR spectrum were acquired in sequence: ^{1}H direct excitation (to detect the liquid phase only), ^{31}P direct excitation without ^{1}H decoupling (to detect the liquid phase only) and ^{31}P direct excitation with ^{1}H decoupling (to detect the liquid and solid phases), with a recycle

delay of 3 s for each type of spectrum. The time to record one sequence of the three spectra was 7.1 min, representing the time-resolution of monitoring the crystallization process in the *in-situ* NMR experiment. Measurement of the sequence of three spectra was repeated throughout the duration of the experiment.

Laboratory Control Synthesis of MFM-500(Ni). BTPPA (0.156 g, 0.29 mmol) and $Ni(NO_3)_2{\cdot}6H_2O$ (0.165 g, 0.57 mmol) were dissolved in D_2O/d_7-DMF (0.5 mL, *v/v* = 1.5:2) at room temperature. The clear green solution was sealed in a vial and heated on an aluminium heating block at 60, 70, 80, 90 and 100 °C until solid formed. The green crystalline product was removed from solution and air dried. **IR:** ν_{max}/ cm^{-1} = 3217, 2170, 1601, 1558, 1408, 1385, 1329, 1250, 1136, 1044, 988, 932, 818, 730.

***Ex-situ* Powder X-ray Diffraction.** For all synthesized samples of green crystalline material, powder XRD data were recorded at 21 °C using a Bruker D8 Diffractometer ($CuK\alpha_1$; Ge-monochromated) operating in transmission mode with a Våntec detector (2θ = 4° - 50°).

***In-situ* Powder X-ray Diffraction.** BTPPA (0.624 g, 1.14 mmol) and $Ni(NO_3)_2{\cdot}6H_2O$ (0.660 g, 2.27 mmol) were dissolved in d_7-DMF (1.14 mL) and D_2O (0.86 mL), respectively, the former requiring mild heating to accelerate dissolution. The solutions were combined at room temperature in a borosilicate glass vial (5 mL), which was placed in the *in-situ* SynRAC cell[6] and heated to 90 °C. Powder XRD data were recorded on beamline I12[7] at Diamond Light Source, Harwell Science and Innovation Campus, UK, using a monochromatic incident beam (λ = 0.23417 Å). Data were recorded at intervals of 4 s on a Thales Pixium RF4343 area detector with 430 × 430 mm^2 active area and a detector distance of 2.402 m. Data processing was carried out using DAWN,[8] with batch Pawley refinements carried out in Topas-Academic V6.[9] Kinetics were modelled in OriginPro 2017 with attempts to use the Gualtieri model[10] fitted to the total integrated diffraction peak area extracted from the Pawley refinements.

Small Angle X-ray Scattering. BTPPA (0.312 g, 0.57 mmol) and $Ni(NO_3)_2{\cdot}6H_2O$ (0.330 g, 1.14 mmol) were dissolved in D_2O and d_7-DMF (1 mL, *v/v* = 1.5:2) at room temperature. The clear green solution was sealed in a vial and heated in an aluminium heating block at 80 °C.

Aliquots were extracted from the solution at different times during the reaction (0, 45, 90, 135, 180, 210 and 240 min; 0 min represents after mixing, but before heating) until green crystals of MFM-500(Ni) were visible by the naked eye. The extracted aliquots were sealed in quartz capillaries and stored at room temperature for a maximum of 2 hr before measurement of SAXS data, which were recorded using a Xenocs Xeuss 2.0 laboratory beamline with a collimated Cu micro-focus source (λ = 1.5406 Å). The sample-to-detector distance was 550 mm, giving scattering vector (Q) in the range 0.02 - 0.72 $Å^{-1}$, where $Q = (4\pi/\lambda)\sin(\theta/2)$. The data were averaged over three consecutive data collections of 600 s.

S2: ^{1}H NMR Peak Assignments in the *In-situ* NMR Study

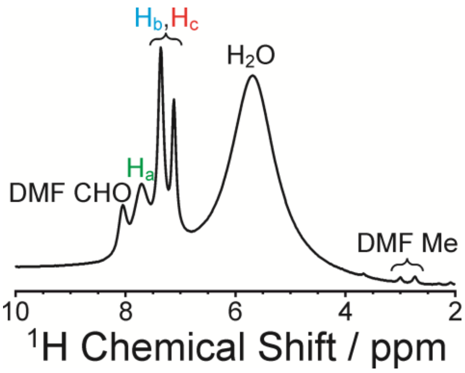

Figure S1. Assignment of the seven ^{1}H NMR resonances observed at all temperatures in the *in-situ* NMR study.

The two weak peaks at lowest chemical shift are assigned to the residual protons in the methyl groups of the solvent d_7-DMF. All spectra were referenced by fixing the ^{1}H chemical shift of the down-field of these two peaks to exactly 3 ppm. The intense, broad peak, initially between 6 and 6.5 ppm at the start of the experiment and then moving to between 4 and 4.5 ppm at the end of the experiment is assigned to water. Although the water in the reaction solvent was initially fully deuterated, the proportion of ^{1}H in the solvent increases over time due to ^{1}H/^{2}H exchange with the acidic linker and with water liberated from $Ni(NO_3)_2 \cdot 6H_2O$. The three peaks observed between 7 and 8 ppm, which move significantly (Figure S2) and decrease in intensity as a function of time, are assigned to the three distinct aromatic ^{1}H environments in the linker. The ratio of integrals of these peaks is 1:2:2; the peak with lowest integral has highest chemical shift and corresponds to the ^{1}H site on the central aryl ring (H_a), while the two peaks of higher integral (H_b, H_c) are due to the two ^{1}H sites on the outer aryl rings (see Figure 1f). The peak at highest chemical shift (*ca*. 8 ppm throughout the experiment) is very weak in the first spectrum of each experiment and is assigned to the aldehyde group of the d_7-DMF solvent. Although initially deuterated, the aldehyde group of d_7-DMF also undergoes ^{1}H/^{2}H exchange with the acidic linker and water as a function of time; as a consequence, the intensity of this peak increases relative to the peaks from the methyl groups of d_7-DMF, which remain weak throughout the experiment.

S3: ^{1}H NMR Peak Shifts of BTPPA During the *In-situ* NMR study

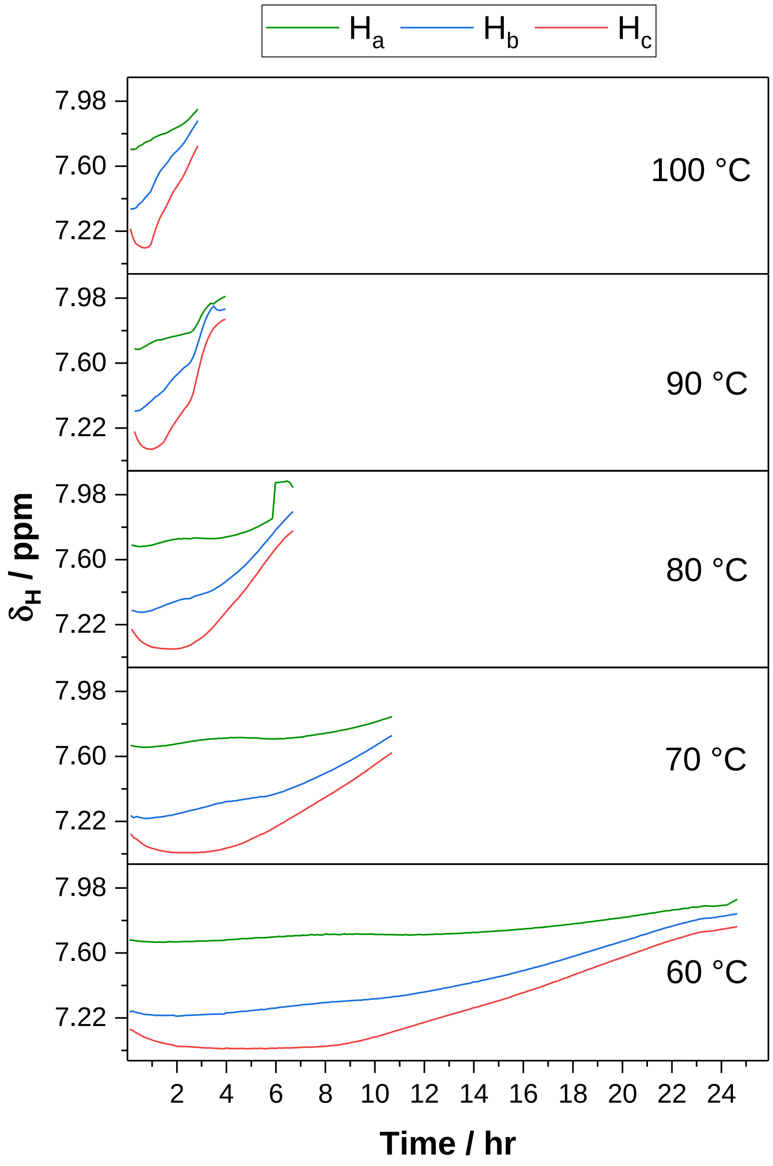

Figure S2. Changes in the positions of the three liquid-state ^{1}H NMR peaks for BTPPA as a function of time during the *in-situ* NMR study at each temperature.

At each temperature, all three ^{1}H NMR signals shift non-monotonically as a function of time during the reaction, initially to lower chemical shift and then to higher chemical shift. The H_c signal exhibits the largest change to lower chemical shift, before increasing to a higher chemical shift. The changes in peak positions are attributed to a combination of deprotonation of the -PO_3H_2 groups, aggregation of linker molecules in solution, and finally complexation of the linkers to Ni(II) ions.

S4: Peak Fitting of ^{1}H NMR Spectra Recorded during the *In-situ* NMR study

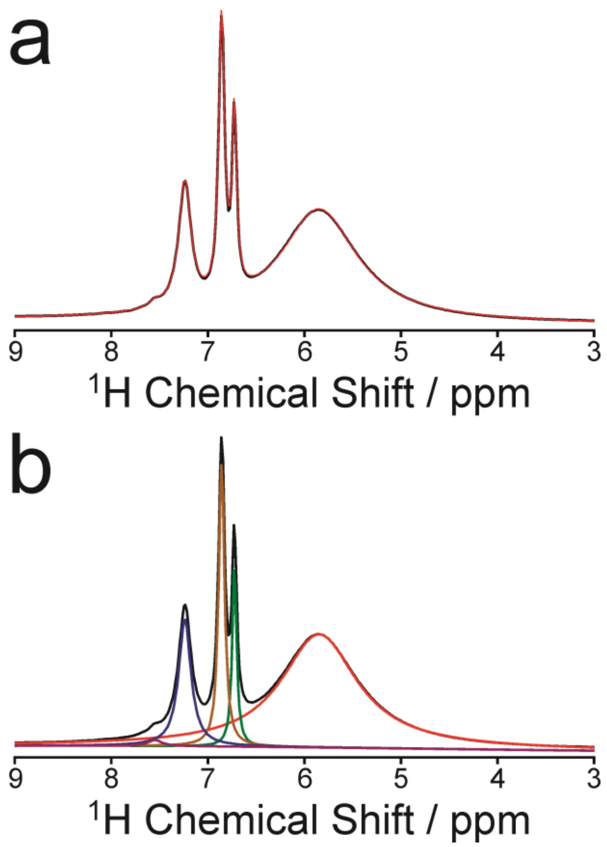

Figure S3. Example of the data fitting of ^{1}H NMR spectra to determine peak intensities, chemical shifts and line widths. (a) The second experimental data set acquired at 80°C (black) and the simulated spectrum from the fitting (red). (b) The same data as in (a) with the five fitted peaks shown separately (in each case, the fitted peak is shown added to the fitted baseline function).

S5: Determination of Kinetic Information from the *In-situ* ^{1}H NMR Data

The ^{1}H NMR spectra recorded as a function of time in the *in-situ* NMR study were fitted to the Gualtieri model described in the main text using Mathematica. The fitted parameters at each temperature are given in Table S1. The fits were carried out several times with different initial values and were found to converge on the values shown.

T / °C	k_n / hr^{-1}	b / hr	k_g / hr^{-1}	s_1	s_2	s_3
60	0.0911(4)	1.809(46)	0.112(2)	0.890(4)	0.887(4)	0.843(4)
70	0.201(1)	0.828(14)	0.226(2)	0.989(3)	0.994(3)	0.981(3)
80	0.301(2)	0.629(14)	0.550(18)	1.021(5)	0.997(5)	0.985(5)
90	0.610(10)	0.400(22)	0.927(54)	1.013(13)	1.012(13)	1.028(13)
100	0.969(9)	0.131(8)	0.848(14)	0.996(10)	0.988(10)	0.973(10)

Table S1. Parameters obtained from fitting the Gualtieri model to the *in-situ* ^{1}H NMR spectra recorded during synthesis of MFM-500(Ni) at each temperature studied. As discussed in the main paper, s_1, s_2 and s_3 are scaling factors. The errors shown are mathematical fitting errors.

In addition to giving the rate constants for nucleation (k_n) and growth (k_g), the model also gives the parameter b (proportional to the standard deviation of $1/k_n$), the variance of the peak defining the distribution of the probability of nucleation with time. The nucleation probability distribution is a *logistic* distribution with mean $1/k_n$ and standard deviation $\sqrt{1/3}\,\pi b$ and is shown in Figure 4(a) for each temperature studied. We note that the nucleation probability distribution is not, as many authors have assumed, a Gaussian distribution. We emphasize that it is the *change* in the intensities of the NMR peaks that provides kinetic information; therefore, quantitative comparison of absolute peak intensities (i.e. between different peaks in a given experiment or comparison between different experiments) is not required to obtain the kinetic information.

S6: NMR Spectra of BTPPA in d_6-DMSO

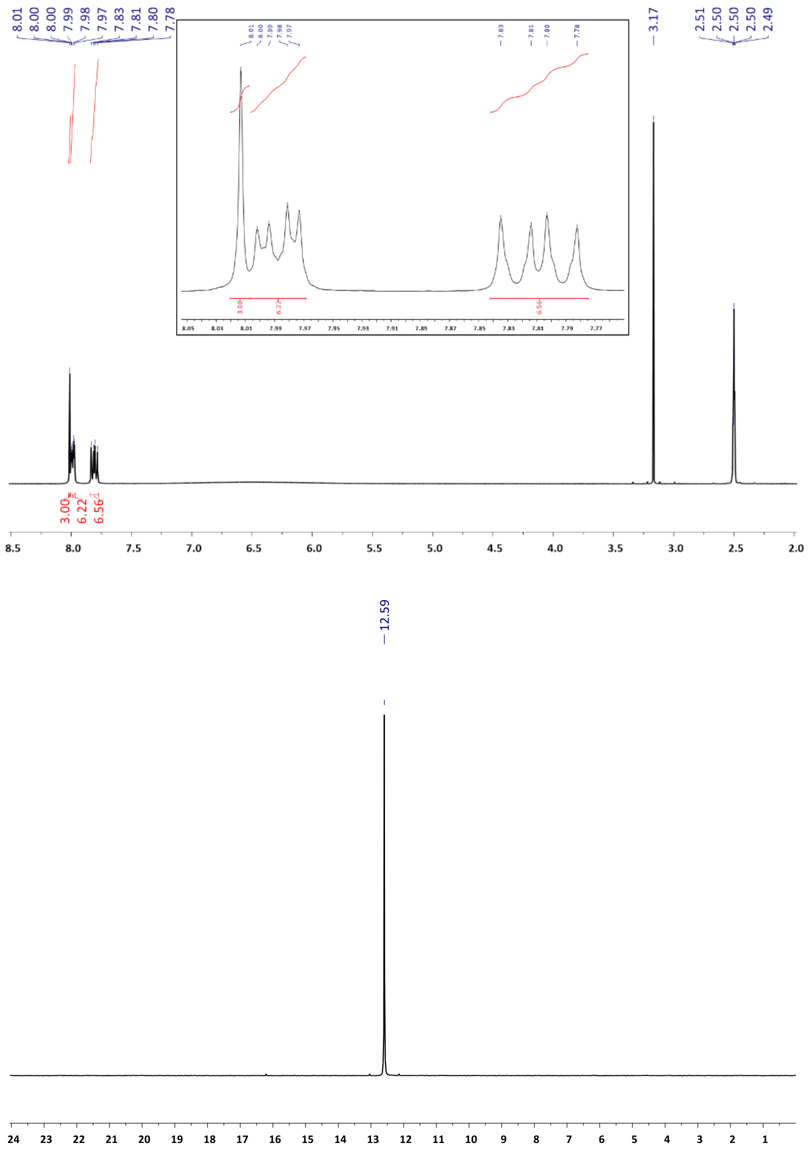

Figure S4. ^{1}H (*top*) and $^{31}P\{^{1}H\}$ (*bottom*) NMR spectra of BTPPA linker in d_6-DMSO.

The BTPPA linker was characterized by ^{1}H and $^{31}P\{^{1}H\}$ NMR (see Figure S4) by dissolving the pale cream solid in d_6-DMSO. The ^{1}H NMR spectrum (room temperature) has three peaks in the aromatic region (see the expanded region), with integrals corresponding to the structure of the linker. The peak at 3.17 ppm is due to residual methanol from the recrystallization step and the quintet at 2.50 ppm is the residual peak due to DMSO. The $^{31}P\{^{1}H\}$ NMR spectrum shows only one ^{31}P environment at 12.59 ppm.

S7: NMR Spectra of BTPPA in D_2O/*d*$_7$-DMF

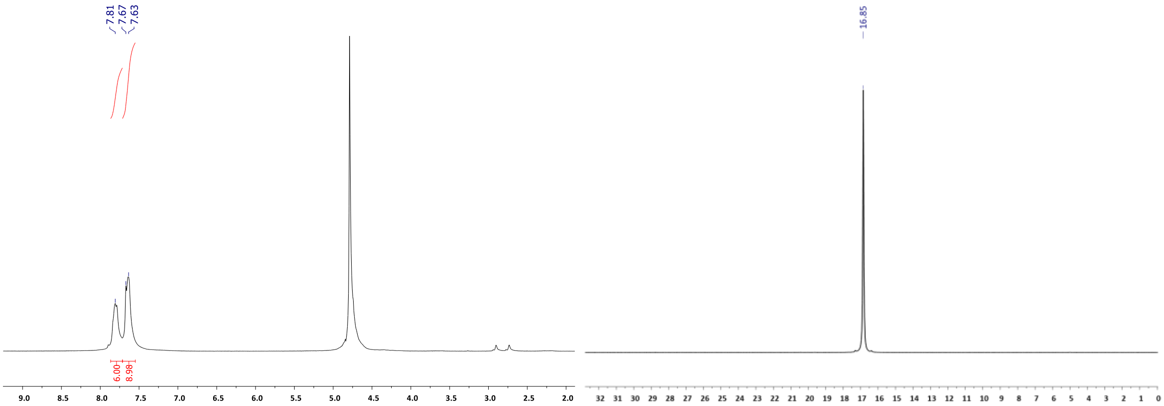

Figure S5. ^{1}H (*left*) and ^{31}P{^{1}H} (*right*) NMR spectra of the BTPPA linker in the reaction solvent mixture (D_2O/*d*$_7$-DMF, *v/v* = 1.5:2).

BTPPA was dissolved in the reaction solution of D_2O and *d*$_7$-DMF (*v/v* = 2:1.5) and a capillary containing D_2O was inserted into the NMR tube as an internal reference. The ^{1}H NMR spectrum (Figure S5) shows only two resolved peaks between 7.5 and 8.0 ppm (in comparison, three aromatic peaks are resolved in Figure S4). One signal integrates to nine protons (i.e., three protons on the central aryl ring overlapping with six protons on the outer aryl rings) and the other signal integrates to six protons (assigned to the rest of the protons on the outer rings). Compared with the ^{1}H NMR spectra recorded in *d*$_6$-DMSO (Figure S4), the peak due to protons on the central aryl ring of the linker is shifted to lower ppm relative to the peaks due to protons on the other aryl rings; furthermore, the peak due to protons on the central aryl ring broadens, suggesting possible aggregation of the linker in solution as the central part of the molecule becomes more shielded from the polar solvent. The small peak at 4.79 ppm is the residual D_2O peak, while the residual DMF peaks are at 2.73, 2.90 and 7.90 ppm. The ^{31}P{^{1}H} NMR spectrum shows a single ^{31}P environment at 16.85 ppm.

S8: Variable Temperature ^{1}H NMR Spectra of BTPPA in D_2O/d_7-DMF

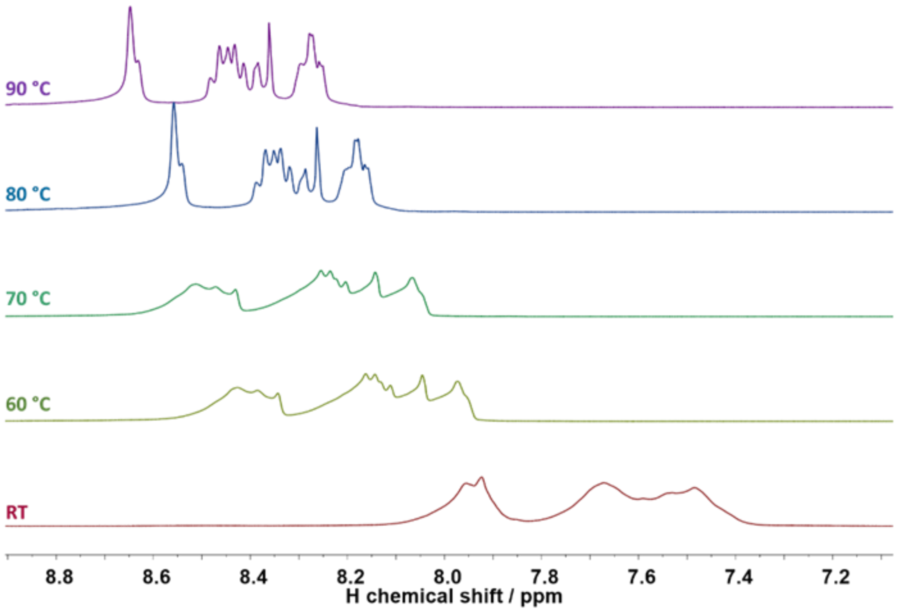

Figure S6. ^{1}H NMR spectra of BTPPA in D_2O/d_7-DMF (spiked with 30 μL of non-deuterated DMF) recorded at room temperature (RT), 60, 70, 80 and 90 °C.

The differences in ^{1}H NMR spectra between BTPPA in pure d_6-DMSO and BTPPA in D_2O/d_7-DMF (i.e., the reaction solvent) suggest that aggregation of the linker occurs in the latter case (see Figures S4 and S5). To investigate the possible aggregation of BTPPA linkers during the reaction, variable temperature ^{1}H NMR spectra were recorded for the reaction solvent D_2O/d_7-DMF spiked with 30 μL of DMF (at natural isotopic abundance) to enable clear identification of the solvent peak. In Figure S6, the ^{1}H NMR peaks for BTPPA are very broad at room temperature. However, as temperature is increased, these peaks move to higher chemical shift and become much narrower, consistent with the occurrence of disaggregation. The reported crystal structure of MFM-500(Ni) contains "dimers" of linkers in a staggered conformation with respect to the three arms of each linker around the central phenyl ring, which are then eclipsed to the next pair of dimers in the "stack" of linkers, resulting in an overall AB…BA…AB motif along the *c*-axis (Figure S7). It is possible that this dimer formation in MFM-500(Ni) originates from aggregation of linkers in solution, as suggested by this NMR

experiment. This possibility is further supported by the SAXS results (see main text and Sections S15 and S16).

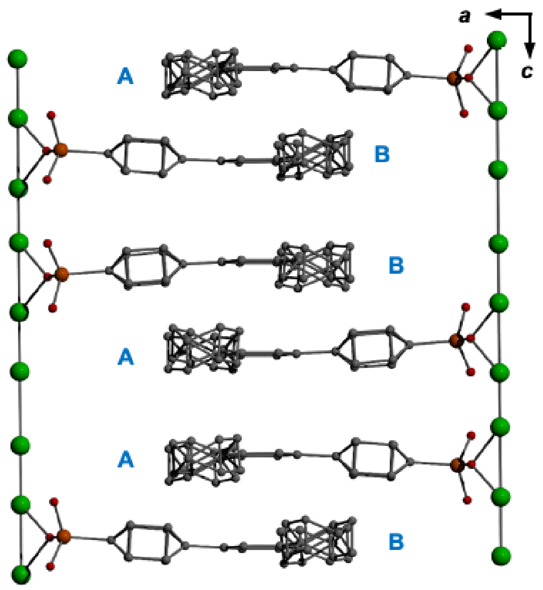

Figure S7. BTPPA linker "dimers" shown with chains of metal ions run in the *c*-direction and the ligands arrange in the AB…BA…AB motif (hydrogen atoms, DMSO molecules and some phosphonic acid groups have been omitted for clarity).

S9: NMR Spectra Recorded *Ex-situ* During Laboratory Synthesis of MFM-500(Ni)

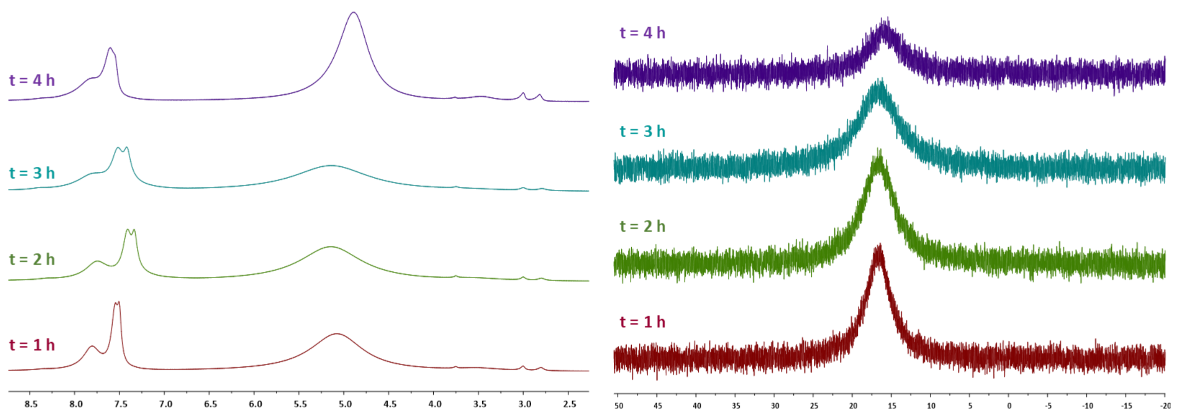

Figure S8. ^{1}H (*left*) and ^{31}P{^{1}H} (*right*) NMR spectra recorded *ex-situ* for samples extracted at different times during laboratory synthesis of MFM-500(Ni) at 80 °C. The NMR spectra were recorded at room temperature.

A reaction of BTPPA and nickel nitrate was set up in a Wheaton vial at 80 °C. An aliquot (0.1 mL) of reaction solution was removed every hour and diluted with D_2O/d_7-DMF (0.3 mL) in the same ratio as the reaction solution. The ^{1}H NMR spectra in Figure S9 show the time series, with each spectrum referenced to the peak at 3.0 ppm (one of the methyl groups in DMF). As time progresses, the aromatic peaks due to the linker move to lower chemical shift, before moving back to higher chemical shift as they merge and diminish in intensity, in agreement with the behaviour observed in the *in-situ* ^{1}H NMR experiment. Furthermore, the intense, broad peak at *ca*. 5.1 ppm also moves as the water environment changes. The ^{31}P{^{1}H} spectra (Figure S8) contain one peak at *ca*. 16 ppm (due to the ^{31}P environment of the linker), which decreases in intensity as the reaction time increases. Although these results are in agreement with the spectra recorded during the *in-situ* NMR experiment at 80 °C, they do not provide enough data points to allow fitting to a kinetic model. Nevertheless, the agreement between these *ex-situ* NMR measurements and our *in-situ* NMR results give strong supporting evidence that the *ex-situ* SAXS measurements at the same temperature (see Section S15) may also be compared directly to the results from our *in-situ* NMR study at 80 °C.

S10: Powder XRD Patterns of the Solid Products Obtained in MFM-500(Ni) Syntheses

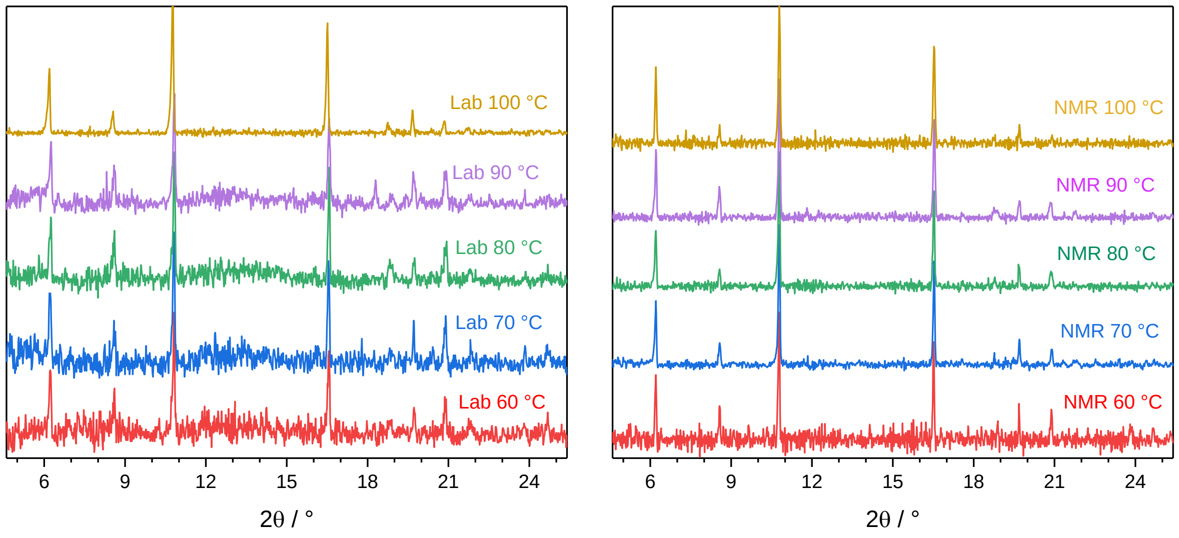

Figure S9. Powder XRD patterns of solid samples recovered from syntheses of MFM-500(Ni) in the laboratory (*left*) and the *in-situ* NMR experiments (*right*).

Powder XRD patterns (Figure S9) recorded for laboratory-synthesized samples of MFM-500(Ni) and for the solid samples recovered from the *in-situ* NMR experiments at 60, 70, 80, 90 and 100 °C are in excellent agreement, confirming that the solid product in all cases was phase-pure MFM-500(Ni).

S11: ^{31}P NMR Spectra Recorded at 60 °C in the *In-situ* NMR study

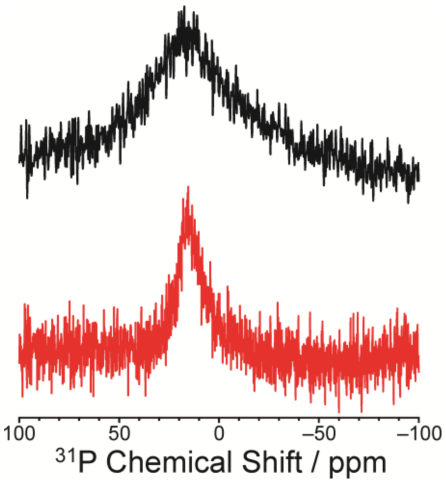

Figure S10. The first direct-excitation ^{31}P NMR spectra recorded after heating to 60 °C in the *in-situ* NMR experiment, without ^{1}H decoupling (*red trace*) and with ^{1}H decoupling (*black trace*).

As shown in Figure S9, the ^{31}P NMR spectra recorded with and without ^{1}H decoupling at the start of the experiment are very broad, even at 60 °C. The signal intensity is lower at the higher temperatures studied in the *in-situ* NMR experiments. Only one signal is observed in the liquid-state ^{31}P NMR spectrum (recorded without ^{1}H decoupling), which diminishes in intensity as a function of time as discussed in the main text (see Figure 2). The first ^{31}P NMR spectrum recorded with ^{1}H decoupling comprises a very broad signal, which also decreases in intensity during the course of the reaction. The reaction concentration is near saturation at room temperature; hence, the observed signal at the start of the experiment is due to incomplete dissolution of solid BTPPA linker as the reaction mixture is heated to 60 °C.

In the ^{31}P NMR spectrum recorded with ^{1}H decoupling, there is no discernible appearance of signals due to the formation of solid MFM-500(Ni) at later times in the experiment. It is possible that the use of a longer recycle delay may have allowed a ^{31}P NMR signal to be observed for solid phases produced in the reaction and this issue will be investigated in future experiments. However, as discussed in section S11, solid-state ^{31}P NMR experiments on a powder sample of MFM-500(Ni) suggest that the recycle delay (3 s) used to record the ^{31}P

NMR spectra with ^{1}H decoupling in our *in-situ* NMR study should be suitable for observing a signal due to the solid MFM-500(Ni) product. While we have no clear explanation for the broadness of the ^{31}P NMR spectra recorded with ^{1}H decoupling in the *in-situ* study, it is conceivable that some amount of paramagnetic Ni may be present in the solid phase formed during the reaction.[11]

S12: Solid-state ^{31}P NMR Spectra for a Powder Sample of MFM-500(Ni)

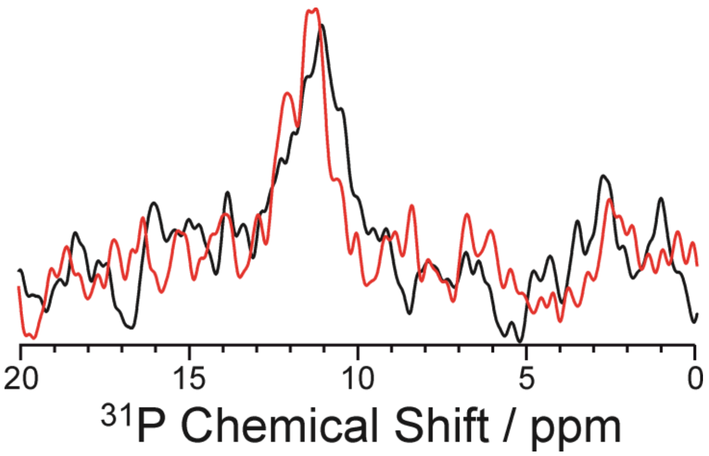

Figure S11. Solid-state ^{31}P NMR spectra acquired for a powder sample of MFM-500(Ni) using ^{31}P direct-excitation and ^{1}H decoupling, with recycle delays of 3 s (black) and 30 s (red). The number of scans in each case was 128.

The recycle delay of 3 s used in our *in-situ* NMR experiments was selected after verifying that solid-state ^{31}P NMR spectra recorded for a powder sample of MFM-500(Ni) using ^{31}P direct-excitation and ^{1}H decoupling gave comparable signal intensity for recycle delays of 3 s and 30 s (Figure S9). We note that solid-state ^{31}P NMR spectra recorded for a powder sample of MFM-500(Ni) using ^{1}H→^{31}P cross-polarization and ^{1}H decoupling did not give any signal. For this reason, ^{1}H→^{31}P cross-polarization was not used within the measurement sequence in our *in-situ* NMR study of the formation of MFM-500(Ni).

S13: Powder XRD Patterns from *In-Situ* Powder XRD

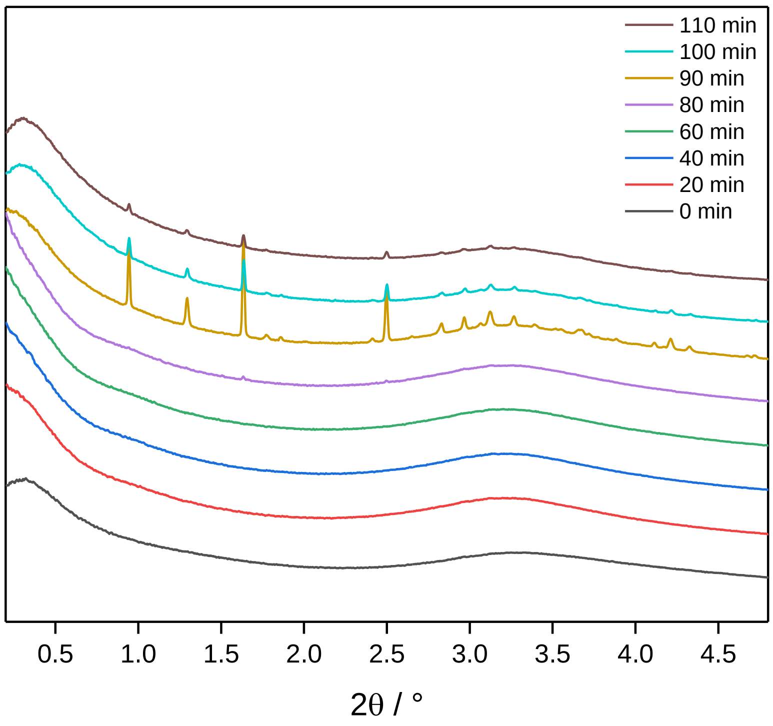

Figure S12. Representative powder XRD patterns recorded during the *in-situ* powder XRD experiment at 90 °C on beamline I12 at Diamond Light Source.

In the *in-situ* powder XRD study at 90 °C, the powder XRD pattern characteristic of MFM-500(Ni) is observed between *ca*. 80 - 90 min (see main text), corresponding to the presence of crystalline particles large enough to be detected by powder XRD. No other crystalline phase was observed throughout the *in-situ* powder XRD experiment (Figure S12).

S14: Attempts to Fit the *In-Situ* Powder XRD Data

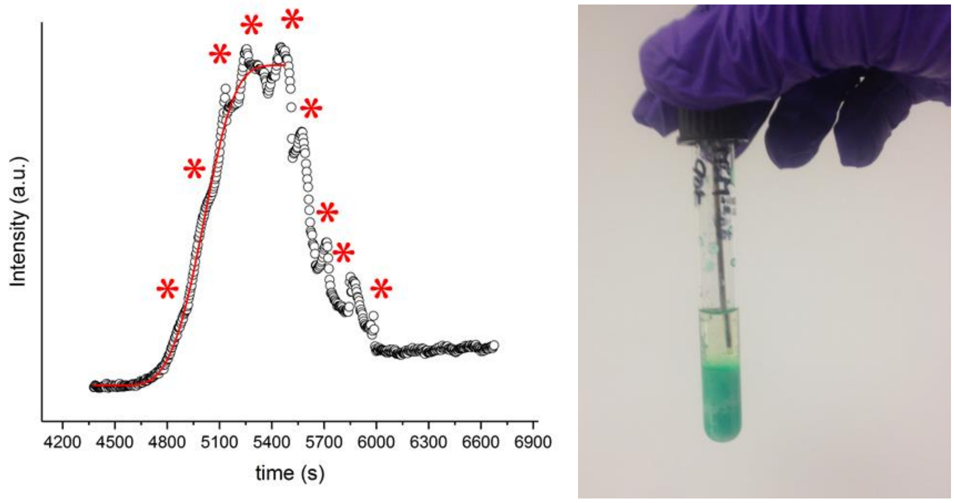

Figure S13. The data shown in the plot (*left*) were produced by carrying out a Pawley refinement on the *in-situ* powder XRD data recorded as a function of time and integrating the area under the diffraction peaks to give a combined “intensity”. The red stars indicate times at which material fell out of the beam path and MFM-500(Ni) collected at the bottom of the sample tube (*right*).

In the results from the *in-situ* powder XRD study (Figure S13), crystalline material is observed around 80 min. However, sudden drops in intensity arise at specific values of time (indicated by red stars in Figure S13). Due to the high concentration of the reaction system, a large amount of solid precipitates and falls unpredictably out of the measurement region of the sample tube (*right*), giving rise to the sudden drops in intensity observed in the plot (*left*). The red trace shows an unsuccessful attempt at a Gualtieri fit.

S15: SAXS Data Recorded *Ex-situ* for Laboratory-synthesized MFM-500(Ni)

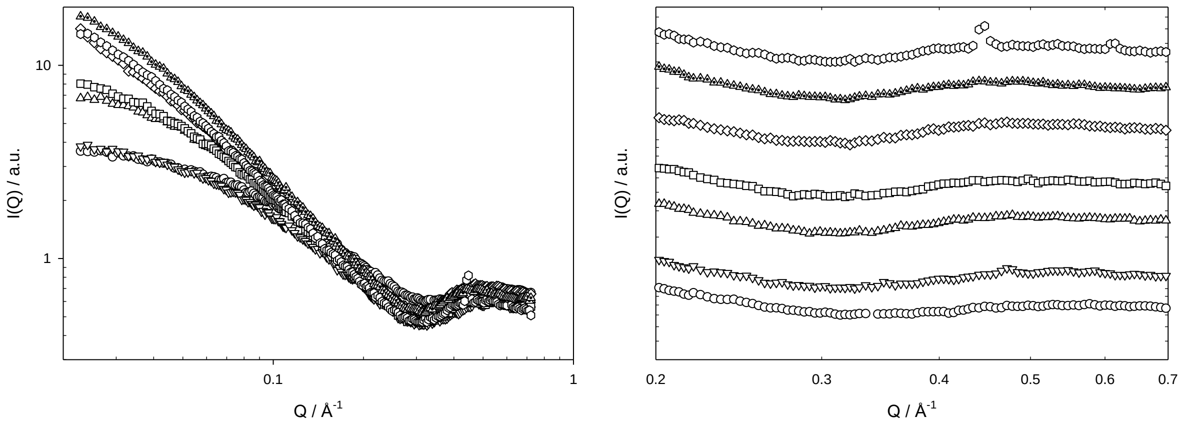

Figure S14: *Left: Ex-situ* SAXS data recorded (at 25 °C) for samples taken from the reaction solution at 80 °C as a function of time: 0 min (before heating, circles), 45 min (inverted triangles), 90 min (triangles), 135 min (squares), 180 min (diamonds), 210 min (dotted triangles) and 240 min (hexagons). *Right:* Expansion of the high *Q* region; data are offset in intensity for clarity.

For all samples, a broad peak centred around Q = 0.5 Å^{-1} is present (Figure S14), corresponding to a length scale of *ca*. 6 Å ($d = 2\pi/Q$). For the sample at 240 min, Bragg peaks are observed at Q = 0.61 Å^{-1} and 0.45 Å^{-1}, corresponding to length scales of 10 Å and 14 Å, respectively, and consistent with peaks in the powder XRD pattern of MFM-500(Ni). At lower Q, the intensity decreases monotonically from 0.02 Å^{-1} to 0.25 Å^{-1}. As time increases, the curvature of the decay decreases, exhibiting $Q^{-1.5}$ dependence at a reaction time of 180 min consistent with the presence of elongated non-spherical species. The intensity of the lower Q region increases as time increases up to 210 min. However, at 240 min, the intensity is lower than at 210 min, since some of the product crystallites are large enough to precipitate and fall out of the measurement region (in a similar manner to the intensity drops observed during the *in-situ* powder XRD experiment above).

S16: Fitting of SAXS Data

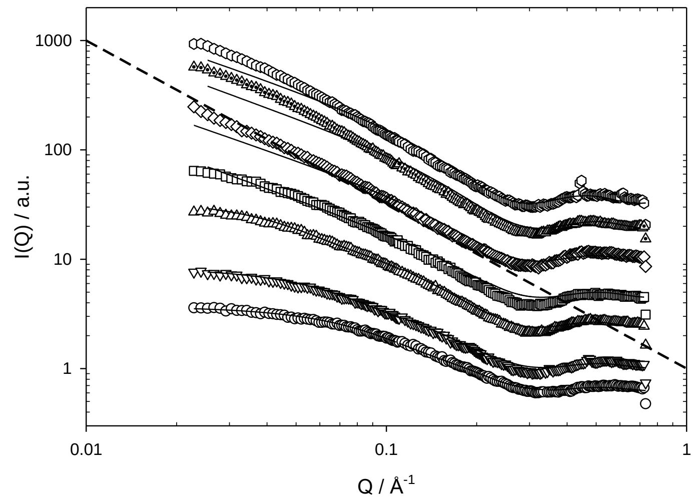

Figure S15: Fitting of the SAXS data recorded *ex-situ* at 0 min (circles), 45 min (inverted triangles), 90 min (triangles), 135 min (squares), 180 min (diamonds), 210 min (dotted triangles) and 240 min (hexagons). For clarity of presentation, measured intensities are scaled by factors of 1, 2, 4, 8, 16, 32 and 64 respectively. Solid lines represent fits to a core-shell cylinder model, as described in the text. The dashed line corresponds to the power law $Q^{-1.5}$.

Data in Figure S15 were analysed by model fitting using the SASview analysis suite of software (version 4.1.2),[12] which uses least-squares analysis to refine model parameters to the data. The data do not fit to a simple model for rods and the best fits are obtained using the form factor for a cylinder with core-shell morphology, which is parameterized by the core radius (r_c), the shell thickness (δ) and the cylinder length (L). The fitted parameters were the core radius and the cylinder length. The shell thickness was fixed at 1 Å to represent a thin region with very different scattering length density to the core, recalling that the SAXS signal is heavily weighted by regions of high electron density and is therefore dominated by the regions occupied by metal atoms. For all samples, the radius of the cylinder was (7.0 ± 0.2) Å (the quoted error is from the fitting process). This radius, combined with the fixed shell width of 1 Å, has similar dimension to the stacks observed in the MFM-500(Ni) crystal structure, with the cylinder walls corresponding to the columns of metal ions that surround the linkers, as

shown schematically on the crystal structure in Figure S16. Such cylindrical stacking is also consistent with the aggregation inferred from NMR data (i.e., Figure S5 and associated text).

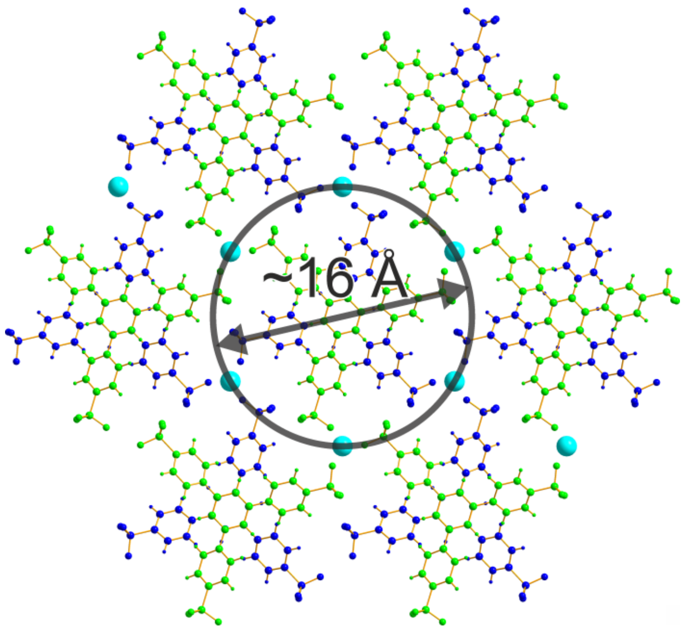

Figure S16: Proposed interpretation of the cylinder with core-shell morphology established from fitting the SAXS data. The crystal structure of MFM-500(Ni) is shown viewed along the *c*-axis.[13] The layer of *green* linkers is in front of a layer of *blue* linkers. It is proposed that the core-shell cylinder model (core radius ≈ 7 Å; shell thickness ≈ 1 Å; total diameter ≈ 16 Å) used to fit the SAXS data corresponds to the black circle region superimposed on the crystal structure, with the metal ions (*large cyan circles*) forming the strongly-scattering walls of the cylinder.

At short reaction times, the core-shell cylinder model gives a reasonable fit to the SAXS data, with the length of the cylinder increasing as the reaction time increases: $L = 52$ Å at $t = 0$ min, $L = 73$ Å at $t = 45$ min, $L = 118$ Å at $t = 90$ min, $L = 880$ Å at $t = 135$ min. From Figure S14, it is clear that, after this time, the model fails to fit the low Q data satisfactorily, presumably because the length scales present are above that accessible for the measured Q range, for which the data are insensitive to lengths of cylinder greater than 900 Å.

In drawing conclusions from this analysis, we recall that the SAXS experiment is heavily weighted towards probing the metal-containing component of the system. The presence of short

cylinders, even at t = 0 min, suggests that structural evolution begins rapidly on mixing, with elongation of the particles occurring up to *ca*. 90 min. At longer times, some particles present are larger than those accessible for the Q range of the experiment. Eventually, precipitation of larger particles leaves a solution of short core-shell cylinders in coexistence with the larger species.

References

1 J. Beckmann, R. Rüttinger and T. Schwich, *Cryst. Growth Des.*, 2008, **8**, 3271–3276.

2 A. Bielecki and D. P. Burum, *J. Magn. Reson. Ser. A*, 1995, **116**, 215–220.

3 A. L. Van Geet, *Anal. Chem.*, 1968, **40**, 2227–2229.

4 A. L. Van Geet, *Anal. Chem.*, 1970, **42**, 679–680.

5 A. E. Aliev and K. D. M. Harris, *Magn. Reson. Chem.*, 1994, **32**, 366–369.

6 N. Heidenreich, U. Rütt, M. Köppen, A. K. Inge, S. Beier, A.-C. Dippel, R. Suren and N. Stock, *Rev. Sci. Instrum.*, 2017, **88**, 104102.

7 M. Drakopoulos, T. Connolley, C. Reinhard, R. Atwood, O. Magdysyuk, N. Vo, M. Hart, L. Connor, B. Humphreys, G. Howell, S. Davies, T. Hill, G. Wilkin, U. Pedersen, A. Foster, N. De Maio, M. Basham, F. Yuan, K. Wanelik and IUCr, *J. Synchrotron Radiat.*, 2015, **22**, 828–838.

8 J. Filik, A. W. Ashton, P. C. Y. Chang, P. A. Chater, S. J. Day, M. Drakopoulos, M. W. Gerring, M. L. Hart, O. V. Magdysyuk, S. Michalik, A. Smith, C. C. Tang, N. J. Terrill, M. T. Wharmby, H. Wilhelm and IUCr, *J. Appl. Crystallogr.*, 2017, **50**, 959–966.

9 A. A. Coelho, *J. Appl. Crystallogr.*, 2018, **51**, 210–218.

10 A. F. Gualtieri, *Phys. Chem. Miner.*, 2001, **28**, 719–728.

11 A. J. Bridgeman, *Dalton Trans.*, 2008, 1989–1992.

12 M. Doucet, J. H. Cho, G. Alina, J. Bakker, W. Bouwman, P. Butler, K. Campbell, M. Gonzales, R. Heenan, A. Jackson, P. Juhas, S. King, P. Kienzle, J. Krzywon, A. Markvardsen, T. Nielsen, L. O'Driscoll, W. Potrzebowski, R. Ferraz Leal, T. Richter, P. Rozycko, T. Snow and A. Washington, DOI:10.5281/ZENODO.825675.

13 S. Pili, S. P. Argent, C. G. Morris, P. Rought, V. García-Sakai, I. P. Silverwood, T. L. Easun, M. Li, M. R. Warren, C. A. Murray, C. C. Tang, S. Yang and M. Schröder, *J. Am. Chem. Soc.*, 2016, **138**, 6352–6355.
